# Prevalence of Metabolic Syndrome and Its Association With Menopausal Symptoms in Post-Menopausal Women: A Scoping Review

**DOI:** 10.7759/cureus.39069

**Published:** 2023-05-16

**Authors:** Vishal Raman, Varsha Kose, Savita Somalwar, Kanchan S Dwidmuthe, Shraddha Rao

**Affiliations:** 1 Department of Obstetrics and Gynecology, NKP Salve Institute of Medical Sciences and Research Centre, Nagpur, IND

**Keywords:** obesity, menopausal symptoms, cardiovascular diseases, post-menopausal women, metabolic syndrome

## Abstract

Complex metabolic dysregulation leads to metabolic syndrome (MetS) causing various symptoms such as type II diabetes, central obesity, cardiovascular diseases (CVD), altered glucose metabolism, hypertension, and dyslipidemia, and is thought to be influenced by a number of factors, including migration from rural to urban areas. socioeconomic changes, and a sedentary lifestyle. Therefore, the primary goal of this scoping review was to determine the prevalence of MetS and its components as well as to understand the association between MetS and menopausal symptoms in post-menopausal women. The search strategy included articles that were published from 2010 onwards in MEDLINE/PubMed, Scopus, and Web of Science databases. The eligibility criteria included population, concept and context (PCC) format and based on it, 10 articles were included in this review. The review concluded that in comparison to pre-menopausal women, MetS is more common in post-menopausal women who are likely to experience somatic complaints and positive correlation of vasomotor symptoms with MetS. Hence, post-menopausal women can be counselled regarding menopausal symptoms related to MetS for which appropriate and adequate treatment or measures should be taken.

## Introduction and background

Metabolic syndrome (MetS) leads to various symptoms that include type II diabetes, central obesity, cardiovascular diseases (CVD), altered glucose metabolism, hypertension, and dyslipidemia which is caused due to complex metabolic dysregulation [1,2]. The incidence of MetS has increased in other South Asian countries as well as in India. MetS can be affected by a number of factors including migration from rural to urban areas, socioeconomic changes, and sedentary lifestyle; the exact etiology of MetS is unknown. The prevalence of MetS varies widely amongst populations, ranging from 13.8% of premenopausal women to 60% of post-menopausal women [3].

During the menopausal transition, reduced estrogen levels and alterations in the testosterone-to-estrogen ratio are directly proportional to the emergence of MetS [4]. Additionally, the increased risk of CVD in post-menopausal women is significantly influenced by changes in lipid metabolism brought on by the deprivation of estrogen levels. Furthermore, studies from all around the world have shown that in post-menopausal women, CVD and MetS are more common [5].

The correlation between MetS and menopausal symptoms has been linked in previous studies [6], while other researchers found that women who reported night sweats or hot flushes had a higher risk of CVD than women who did not have these symptoms [7]; Some studies reported that an increased risk of hot flushes is related to higher abdominal adiposity [6]. In a different research, it was discovered that patients with and without MetS had the same vasomotor symptoms [8]. Numerous studies have also examined the relationships between psychological signs and symptoms and MetS [9]. As a result, the primary objective of this scoping review was to determine the prevalence of MetS and its components and to understand the relationship between menopausal symptoms and MetS in post-menopausal women.

## Review

This review followed Preferred Reporting Items for Systematic Review and Meta-Analysis extension for Scoping Review (PRISMA-ScR) guidelines. The eligibility criteria included population, concept, and context (PCC) format in which population involving all post-menopausal women attending the Department of Obstetrics and Gynaecology and were willing to participate in the study were included in this review; women on hormone replacement therapy, having primary hypertension, type I diabetes mellitus, thyroid disorders, and congenital obesity were excluded from this review. Literature elaborating on the application of the concept of MetS in post-menopausal women and its association with symptoms was included in this review. The context format consisted of literature or studies done in tertiary health care centres in any country; whereas, literature or studies related to primary or secondary health care systems were excluded from this review.

Additionally, all the studies published from 2010 onwards that were in the English language were included in this review; whereas, articles published before 2010 and that were not in the English language were excluded from this review. The articles with prospective cross-sectional, exploratory, cohort, and observational study designs consisting of quantitative, qualitative, and mixed methods were included in this review. Research related to pregnant women, case reports, case series, duplicate studies, and animal studies were excluded.

Search strategy

Articles that were published from 2010 onwards in MEDLINE/PubMed, Scopus, and Web of Science databases were included in the search strategy. The keywords that were used to search consisted of “metabolic syndrome”, “pre-menopausal women”, “post-menopausal women”, “menopausal symptoms” and “prevalence”. Boolean operators consisting of “metabolic syndrome” AND “pre-menopausal women” AND “post-menopausal women” AND “prevalence” AND “menopausal symptoms”.

Study selection

**Figure 1:**
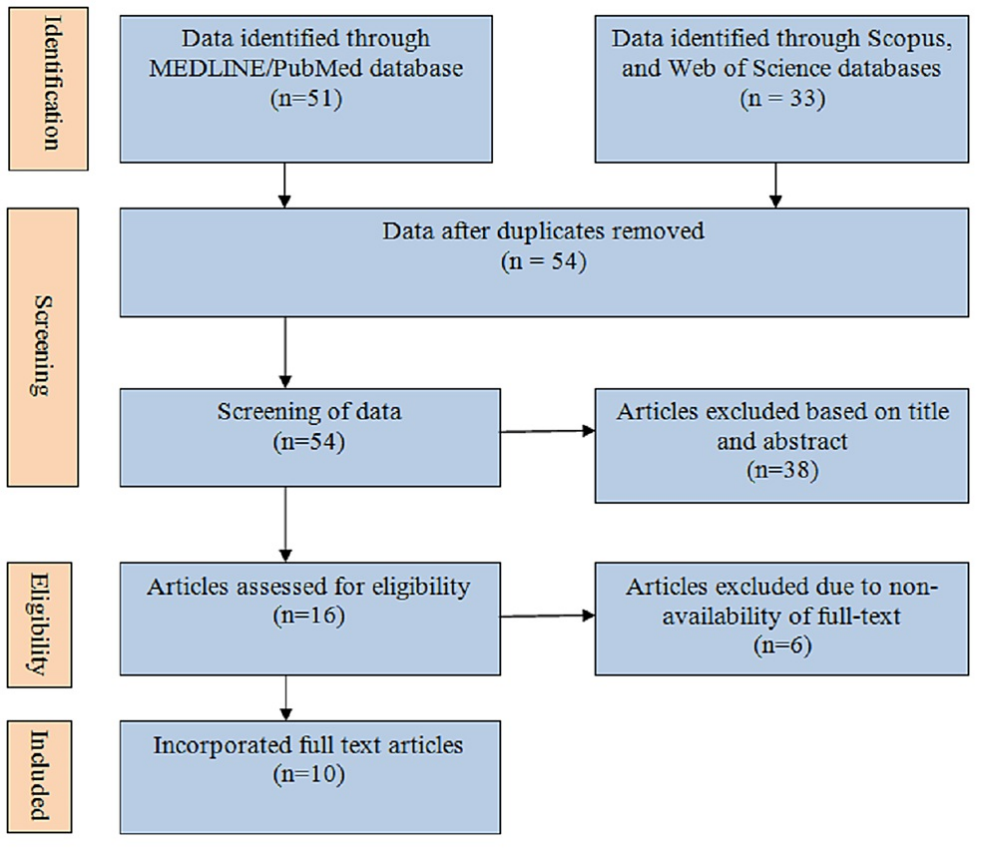
Search strategy.

Data identified through MEDLINE/PubMed, Scopus, and Web of Science databases consisted of 84 articles out of which 30 were duplicates, considering overall 54 articles that were screened. Out of 54 articles, 38 articles were removed based on title and abstract, and all 16 articles were assessed for eligibility. Articles excluded due to non-availability of full-text consisted of six articles and finally 10 full-text articles were incorporated for the present scoping review in which the frequency of MetS in post-menopausal women was studied (Figure 1).

Data extraction

Particular details associated with context, population, and concept together with the name of the authors of the study, study design, aim of the study, summary of findings, and participant attributes were mentioned in all the 10 studies and were incorporated in this review (Table 1).

**Table 1:** Details of the articles included in the review. MetS = metabolic syndrome.

Sr. No.	Author and year	Study design	Aim	Participants	Summary of findings
1	Mehndiratta et al. 2020 [3]	Prospective cross-sectional study	To determine whether pre-and post-menopausal women have higher rates of the MetS.	Total 200 patients	Postmenopausal women were shown to have a greater incidence of MetS.
2	Cengiz et al. 2019 [8]	Cross-sectional study	To investigate the connection between post-menopausal women's MetS and menopausal symptoms	Total 541 patients	The study came to the conclusion that the existence of MetS was linked to a number of menopausal symptoms.
3	Jeyasheela et al. 2018 [10]	Prospective cross-sectional study	To check if postmenopausal women are more likely to have the MetS.	Total 150 patients	Due to oestrogen shortage and fat redistribution, post-menopausal women have a higher incidence of MetS.
4	Marchi et al. 2017 [11]	Prospective cross-sectional study	To determine MetS and its symptoms in pre-and post-menopausal women.	Total 958 patients	In comparison to pre-menopausal women, MetS is more common in Post-menopausal women.
5	Bharath et al. 2020 [12]	Descriptive study	To determine whether MetS is more common in post-menopausal women.	Total 104 patients	Among post-menopausal women, MetS is very common.
6	Sharma et al. 2016 [13]	Cross-sectional study	To find out how common MetS is among North Indian women who are pre- and postmenopausal.	Total 350 patients	In older and obese women, the prevalence of MetS was shown to be greater.
7	Ryu et al. 2015 [14]	Cross-sectional study	To determine whether postmenopausal women's vasomotor symptoms are associated with a higher risk of developing MetS.	Total 1906 patients	Vasomotor symptoms are positively correlated with MetS.
8	Lee et al. 2011 [6]	Cross-sectional study	To ascertain the relationship between MetS and menopausal symptoms.	Total 183 women	Women with MetS are more likely to experience somatic complaints.
9	Yoldemir et al. 2012 [15]	Cross-sectional study	To find out whether post-menopausal and pre-menopausal women have more MetS than either group.	Total 180 women	In comparison to pre-menopausal women, MetS did not differed much in post-menopausal women.
10	Pandey et al. 2010 [16]	Cross-sectional study	To determine whether MetS is more common in postmenopausal women when compared to pre-menopausal women.	Total 498 women	In comparison to pre-menopausal women, MetS is more common in Post-menopausal women.

Numerical demographic data consisting of post-menopausal MetS, age, waist circumference, waist-to-hip ratio, body mass index, systolic blood pressure, diastolic blood pressure, high-density lipoprotein, low-density lipoprotein, triglycerides, and fasting blood glucose parameters are demonstrated in Table 2.

**Table 2:** Numerical demographic data of the studies included. BMI = body mass index, HDL = high-density lipoprotein, LDL = low-density lipoprotein.

Author and Year	Metabolic Syndrome (Post-menopausal)	Age	Waist circumference (cm)	Waist-to-hip ratio	BMI	Systolic blood pressure (mmHg)	Diastolic blood pressure (mmHg)	HDL (mg/dl)	LDL (mg/dl)	Triglycerides (mg/dl)	Fasting glucose (mg %)
Mehndiratta et al. 2020 [3]	42 (42.0%)	52.40±4.155	86.00±9.14	0.93±0.05	_	140.72±19.83	88.40±11.82	46.00±12.2	_	169.52±43.88	46 (46)
Jeyasheela et al. 2018 [10]	99 (64%)	58.22±7.2	95.63±9.32	_	28.19±5.38	134.71±19.53	78.91±12.15	49.60±18.605	114.91±36.09	136.45±70.94	129.48±56.35
Marchi et al. 2017 [11]	151 (22.2%)	57.3 ± 5.33	102 (76.7)	_	77 (73.3)	468 (77.7)	_	249 (77.8)	166 (74.4)	146 (78.5)	154 (82.8)
Bharath et al. 2020 [12]	65 (62.5%)	55.34±7.27	89.22±8.04	_	25.68±2.67	131.28±16.91	84.8±11.21	47.8±9.49	_	168.85±74.05	112.18±30.94
Sharma et al. 2016 [13]	115 (65.7%)	49.54 ± 2.724	_	0.93 ± 0.05	37 (46.3%)	119 (68%)	79 (45.1)	95 (54.9)	_	91 (52%)	52 (29.7)
Ryu et al. 2015 [14]	245 (22.2%)	53 (45-65)	82.5 (61.7-130.7)	_	82.5 (61.7-130.7)	110 (70-170)	70 (40-100)	55 (8-109)	100 (40-194)	96 (33-742)	92 (72-204)
Lee et al. 2011 [6]	_	56.4 ± 5.7	_	0.92 ± 0.04	25.5 ± 2.5	131.5 ± 17.3	82.9 ± 9.2	43.6 ± 10.4	124.6 ± 36.5	184.1 ± 63.7	99.3 ± 18.7
Yoldemir et al. 2012 [15]	_	57.49 ± 5.78	_	0.88 ± 0.09	29.83 ± 4.06	128.29 ± 26.25	91.06 ± 25.22	47.17± 20.47	139.66 ± 38.40	199.91 ± 78.66	119.74± 40.83
Pandey et al. 2010 [16]	224 (45.0%)	55.91 ± 7.58	89.43 ± 10.91	_	27.70 ± 4.70	131.12 ± 18.62	80.83 ± 7.97	49.31 ± 11.12	134.39 ± 34.81	146.55 ± 86.06	89.44 ± 25.70
Cengiz et al. 2019 [8]	_	55.59±6.75	_	_	30.20±5.24	128.66±18.39	79.68±10.31	1.66±0.07	_	2.22±0.18	107.98±33.64

Discussion

This scoping review aimed to evaluate the prevalence of MetS and its components as well as the relationship between MetS and menopausal symptoms in post-menopausal women. The review involved 10 articles that concluded that MetS is more common in post-menopausal women and are more likely to experience somatic complaints and a positive correlation of vasomotor symptoms with MetS, in comparison to pre-menopausal women. This is due to estrogen shortage and fat redistribution. Additionally, the incidence of MetS was shown to be more in obese and older women.

In a study presented by Mehndiratta et al., it was discovered that the incidence of MetS was more in post-menopausal women accounting for 42% as compared to the premenopausal group which was 16% [3]. The outcomes were moderately consistent with the study given by Ahuja [17] which demonstrated that MetS was found in 28% of the study group, with 21% of the patients being postmenopausal and 7% being premenopausal. Similarly, Toppo et al. [18] discovered that 38% of premenopausal and postmenopausal women had MetS overall. Additionally, Pandey et al. revealed that MetS among post-menopausal women over the age of 35 years is more common [16]. Whereas in a study given by Yoldemir et al., a negligible difference between pre-menopausal and post-menopausal women regarding MetS was reported [15].

When various components of the MetS were determined, the highest incidence was for hypertension accounting for 58%, followed by increased waist circumference consisting of 42%, high triglyceride levels at 40%, low-density lipoprotein (LDL) consisting of 37%, and diabetes at 34% which was moderately consistent with the research conducted by Gupta et al. [19] in which the most common component of MetS was hypertension (51%), followed by waist circumference accounting for 34%, triglyceride consisting of 33%, and diabetes with 17%.

According to Cengiz et al., menopausal symptoms involving sleeping problems, physical and mental fatigue, voiding issues, obesity, irritability, depressive mood, and anxiety are all associated with MetS [8]. Additionally, Lee et al. observed in their study that somatic symptoms including sweating and hot flushes were more common in the MetS group and had a positive correlation with serum triglyceride levels [6]. Similar findings were made by Chedraui et al. in their study, which demonstrated that a substantial risk factor for depression, hot flushes, and joint pain is abdominal obesity [20].

According to research by Jeyasheela et al., 64% of postmenopausal women have MetS, along with larger waist circumference, and systolic blood pressure [10]. According to Heidari et al., the prevalence of MetS in premenopausal women was 44.9%, in perimenopausal was 57.9%, and in postmenopausal women was 64.3% respectively, in a study comprising 1596 women [21]. According to Misra et al., Asian Indians appear to have reduced CVD than Caucasians due to their lower waist circumference levels [22]. This is because, despite similar or slightly lower average values for waist circumference, Africans and Caucasians have considerably higher fat mass over the trunk and abdomen. Asian Indians may have higher levels of insulin resistance and other CVD risk factors than Caucasians, despite having lower levels of truncal and abdominal obesity [23,24].

According to Marchi et al., postmenopausal women showed a higher prevalence of MetS which also progresses with age. Postmenopausal women were more likely to have low HDL-C levels, hypertension, and high fasting blood sugar (FBS) levels which were all symptoms of MetS [11]. Similar elements were common in earlier investigations carried out in Iran [25,26], and South Korea [27]. Similarly to this, Bharath et al. reported in their study that post-menopausal women had a 62.5% prevalence of MetS [12]. The components of MetS such as blood pressure, FBS, triglycerides, and waist circumference were significantly raised and HDL levels were significantly reduced which is congruous with the previous studies [10,28].

Sharma et al. also noted in their study that there is a very high prevalence of MetS in North Indian women, which is related to the greater prevalence of obesity in this population. The study also emphasized the connections between different MetS components and their prevalence. The highest correlation between MetS risk factors and HDL was discovered, while the lowest association was discovered with diabetes [13]. These findings were consistent with earlier research by Jesmin et al., which discovered a strong link between the occurrence of MetS and altered HDL levels [29], however, Afzal and Bashir's investigation found that hypertension and MetS had the highest association followed by obesity [30]. Additionally, Ryu et al. reported that Korean postmenopausal women had vasomotor symptoms linked to MetS and the key metabolic factors related to vasomotor symptoms include obesity and lipid abnormalities [14] and therefore it had a greater triglyceride to HDL-C ratio [31] which is a significant characteristic of MetS.

As women with MetS have abdominal obesity and more body fat, an increase in vasomotor symptoms is caused by an obstruction of heat dissipation, a decrease in core body temperature, and increase in body fat [6]. In relation to this, a significant risk factor for muscle and joint pain, depression, and hot flushes is abdominal obesity; whereas, changes in sexual desire, and dry skin were associated with basal hyperglycemia, and increased sweating and depression were related to high triglyceride levels [20]. Therefore, because of the morbidity and mortality linked to MetS, it is necessary to regularly screen for and diagnose the condition in order to prevent its repercussions.

## Conclusions

This review concludes that MetS is more common in post-menopausal women and are more likely to experience somatic complaints and a positive correlation of vasomotor symptoms with MetS, in comparison to pre-menopausal women which is due to estrogen shortage and fat redistribution. Additionally, in older and obese women, the prevalence of MetS was shown to be greater. These consequences linked to MetS will reduce with early diagnosis and rapid treatment. Hence, post-menopausal women can be counselled regarding menopausal symptoms related to MetS for which appropriate and adequate treatment or measures should be taken.

## References

[1] Carr MC (2003). The emergence of the metabolic syndrome with menopause. J Clin Endocrinol Metab.

[2] Reckelhoff JF, Fortepiani LA (2004). Novel mechanisms responsible for postmenopausal hypertension. Hypertension.

[3] Mehndiratta N, Sharma S, Sharma RK, Grover S (2020). A prospective study on the incidence of metabolic syndrome in premenopausal and postmenopausal women. J Midlife Health.

[4] Mesch VR, Boero LE, Siseles NO (2006). Metabolic syndrome throughout the menopausal transition: influence of age and menopausal status. Climacteric.

[5] Sapkota AS, Sapkota A, Acharya K, Raut M, Jha B (2015). Study of metabolic syndrome in postmenopausal women. Clin Chem Lab Med.

[6] Lee SW, Jo HH, Kim MR, Kwon DJ, You YO, Kim JH (2012). Association between menopausal symptoms and metabolic syndrome in postmenopausal women. Arch Gynecol Obstet.

[7] Zhu D, Chung HF, Dobson AJ (2020). Vasomotor menopausal symptoms and risk of cardiovascular disease: a pooled analysis of six prospective studies. Am J Obstet Gynecol.

[8] Cengiz H, Kaya C, Suzen Caypinar S, Alay I (2019). The relationship between menopausal symptoms and metabolic syndrome in postmenopausal women. J Obstet Gynaecol.

[9] Grundy SM, Cleeman JI, Daniels SR (2005). Diagnosis and management of the metabolic syndrome: an American Heart Association/National Heart, Lung, and Blood Institute Scientific Statement. Circulation.

[10] Jeyasheela K, Ebenezer ED, Londhe V, Paul TV, Yadav B, Kekre AN (2018). Prevalence of metabolic syndrome among postmenopausal women in South India. Int J Reprod Contracept Obstet Gynecol.

[11] Marchi R, Dell'Agnolo CM, Lopes TC (2017). Prevalence of metabolic syndrome in pre- and postmenopausal women. Arch Endocrinol Metab.

[12] Bharath G, Itagi A, Mahendra SV (2020). Assessment of occurrence of metabolic syndrome among post-menopausal women. J Adv Med Med Res.

[13] Sharma S, Aggarwal N, Joshi B, Suri V, Badada S (2016). Prevalence of metabolic syndrome in pre- and post-menopausal women:a prospective study from apex institute of North India. J Midlife Health.

[14] Ryu KJ, Park HT, Kwon DH (2015). Vasomotor symptoms and metabolic syndrome in Korean postmenopausal women. Menopause.

[15] Yoldemir T, Erenus M (2012). The prevalence of metabolic syndrome in pre- and post-menopausal women attending a tertiary clinic in Turkey. Eur J Obstet Gynecol Reprod Biol.

[16] Pandey S, Srinivas M, Agashe S, Joshi J, Galvankar P, Prakasam CP, Vaidya R (2010). Menopause and metabolic syndrome: a study of 498 urban women from western India. J Midlife Health.

[17] Ahuja M (2016). Age of menopause and determinants of menopause age: a pan India survey by IMS. J Midlife Health.

[18] Toppo A, Varma S, Sahu L (2017). Comparative evaluation of metabolic syndrome in premenopausal and postmenopausal women. IOSR J Dent Med Sci.

[19] Gupta R, Sarna M, Thanvi J, Rastogi P, Kaul V, Gupta VP (2004). High prevalence of multiple coronary risk factors in Punjabi Bhatia community: Jaipur Heart Watch-3. Indian Heart J.

[20] Chedraui P, Hidalgo L, Chavez D, Morocho N, Alvarado M, Huc A (2007). Menopausal symptoms and associated risk factors among postmenopausal women screened for the metabolic syndrome. Arch Gynecol Obstet.

[21] Heidari R, Sadeghi M, Talaei M, Rabiei K, Mohammadifard N, Sarrafzadegan N (2010). Metabolic syndrome in menopausal transition: Isfahan Healthy Heart Program, a population based study. Diabetol Metab Syndr.

[22] Misra A, Vikram NK, Gupta R, Pandey RM, Wasir JS, Gupta VP (2006). Waist circumference cutoff points and action levels for Asian Indians for identification of abdominal obesity. Int J Obes (Lond).

[23] Chandalia M, Abate N, Garg A, Stray-Gundersen J, Grundy SM (1999). Relationship between generalized and upper body obesity to insulin resistance in Asian Indian men. J Clin Endocrinol Metab.

[24] Vikram NK, Pandey RM, Misra A, Sharma R, Devi JR, Khanna N (2003). Non-obese (body mass index < 25 kg/m2) Asian Indians with normal waist circumference have high cardiovascular risk. Nutrition.

[25] Eshtiaghi R, Esteghamati A, Nakhjavani M (2010). Menopause is an independent predictor of metabolic syndrome in Iranian women. Maturitas.

[26] Ainy E, Mirmiran P, Zahedi Asl S, Azizi F (2007). Prevalence of metabolic syndrome during menopausal transition Tehranian women: Tehran Lipid and Glucose Study (TLGS). Maturitas.

[27] Siemińska L, Wojciechowska C, Foltyn W (2006). The relation of serum adiponectin and leptin levels to metabolic syndrome in women before and after the menopause. Endokrynol Pol.

[28] Jouyandeh Z, Nayebzadeh F, Qorbani M, Asadi M (2013). Metabolic syndrome and menopause. J Diabetes Metab Disord.

[29] Jesmin S, Islam AM, Akter S (2013). Metabolic syndrome among pre- and post-menopausal rural women in Bangladesh: result from a population-based study. BMC Res Notes.

[30] Afzal S, Bashir MM (2008). Prevalence of metabolic syndrome in pre and postmenopausal diabetics. Biomedica.

[31] Reaven G (2002). Metabolic syndrome: pathophysiology and implications for management of cardiovascular disease. Circulation.

